# More than pretty pictures? How illustrations affect parent-child story reading and children's story recall

**DOI:** 10.3389/fpsyg.2014.00738

**Published:** 2014-07-22

**Authors:** Andrea Follmer Greenhoot, Alisa M. Beyer, Jennifer Curtis

**Affiliations:** ^1^Department of Psychology, University of KansasLawrence, KS, USA; ^2^Department of Psychology, Northern Arizona UniversityYuma, AZ, USA

**Keywords:** children's memory, parent-child story reading, illustrations, preschoolers, story recall

## Abstract

Previous research showed that story illustrations fail to enhance young preschoolers' memories when they accompany a pre-recorded story (e.g., Greenhoot and Semb, 2008). In this study we tested whether young children might benefit from illustrations in a more interactive story-reading context. For instance, illustrations might influence parent-child reading interactions, and thus children's story comprehension and recall. Twenty-six 3.5- to 4.5-year-olds and their primary caregivers were randomly assigned to an Illustrated or Non-Illustrated story-reading condition, and parents were instructed to “read or tell the story” as they normally would read with their child. Children recalled the story after a distracter and again after 1 week. Analyses of the story-reading interactions showed that the illustrations prompted more interactive story reading and more parent and child behaviors known to predict improved literacy outcomes. Furthermore, in the first memory interview, children in the Illustrated condition recalled more story events than those in the Non-Illustrated condition. Story reading measures predicted recall, but did not completely account for picture effects. These results suggest that illustrations enhance young preschoolers' story recall in an interactive story reading context, perhaps because the joint attention established in this context supports children's processing of the illustrations.

## Introduction

A glance at the early childhood sections of any library or bookstore reveals that pictures books, or books in which pictures complement or dominate the text (Jalongo, 2004), are quite common in young children's literature. Why are illustrations so ubiquitous in story books for young children? It is widely believed that story illustrations help capture children's attention to stories and facilitate their understanding and retention of what is being read to them. This conclusion is bolstered by studies of preschool children's visual attention during storybook reading, which shows that they are overwhelmingly focused on the illustrations rather than the print (e.g., Evans and Saint-Aubin, 2005; Justice et al., 2008). Although pictures may capture children's attention, research from our laboratory and others suggests that they may not actually enhance very young children's comprehension and recall of the stories they accompany, at least in controlled story presentation contexts (e.g., Greenhoot and Semb, 2008). It remains to be seen, however, whether illustrations have different effects on children's processing of stories when they are read in a more naturalistic and interactive story-reading context. Therefore, we designed this experiment to examine the effects of story illustrations on parent-child story reading and preschool children's story recall. Because our work is grounded in the literature on memory and narrative development, we focused on children's abilities to recall the major events that took place in the story, rather than other dimensions such as vocabulary or a moral.

It is well-established that, among school-aged children and adults, memory for prose that is presented in written or auditory form is enhanced by illustrations (Levin and Lesgold, 1978; Brookshire et al., 2002; Carney and Levin, 2002). There are a number of explanations for this picture-enhancement effect. Exposure to information both verbally and pictorially may result in the construction of memory representations in both modalities that then provide redundant retrieval routes (Paivio, 1986, 1970). Pictures may also enhance attention and comprehension or organization of material, or provide cues about important information in the text to keep activated, all which may promote the formation of stronger, more elaborated and more organized memory traces (Gernsbacher, 1990; Levin and Mayer, 1993).

Although picture-facilitation effects are well-established in the literature on school-aged children and adults, the developmental literature suggests that story illustrations might not yield the same benefits for very young children as have been observed for older children (e.g., Guttman et al., 1977; Furnham et al., 2002; Pike et al., 2010). A few studies have documented picture-enhancement effects in preschoolers but only for very specific auditory information (e.g., the object of a sentence) that is also explicitly depicted in the pictures (Pressley et al., 1982; Digdon et al., 1985). This line of work has also shown that younger children require greater redundancy between the pictures and auditory information to show mnemonic benefits than do older children (Guttman et al., 1977; Furnham et al., 2002).

Research from our own laboratory on children's memories for more complex story narratives found that illustrations failed to enhance the memories of young preschoolers when they accompanied prerecorded stories (Greenhoot and Semb, 2008). In that study, children who were between 46 and 63 months of age were exposed to a story about a fictional animal character in one of four story-presentation formats: the verbal story narrative with illustrations, the narrative alone, the narrative with uninformative illustrations, or the illustrations alone. To ensure that the verbal presentation was identical across groups, the story narrative was pre-recorded and a tone cued children to turn the page. Although children in all verbal groups accurately recalled more story events than those in the picture-only group, for children younger than 4.5 years, there were no differences in recall performance between the three verbal groups. With increasing age, children exposed to the illustrated story narrative increasingly outperformed those in the other verbal groups, such that children older than 4.5 years did benefit from the informative illustrations. These benefits, moreover, were limited to information presented both in the text and the pictures. The overall pattern of results suggested that the illustrations did not simply improve motivation and attention to the listening task. Rather, the children must have attended to the content of the pictures because it determined whether they were effective in facilitating recall.

One explanation for the younger children's failure to benefit from story illustrations is that they may not understand the relevance of illustrations and therefore fail to encode the illustrations or use them as retrieval cues (e.g., Pressley and MacFadyen, 1983). Indeed, in the handful of studies that observed picture facilitation effects for preschoolers' recall of simple stimuli, the children were warned of the memory test and explicitly prompted to attend to the pictures (Pressley et al., 1982; Digdon et al., 1985). Another possibility is that very young children lack the processing capacity necessary to attend to and encode both the story and the pictures and to connect them in memory (Dempster, 1981; Cowan et al., 2002). Consistent with this argument, Mayer and Moreno (1998) showed that adults' ability to combine auditory and visual details in memory depends on the availability of working memory resources. Finally, the literature on symbolic development would suggest that young children struggle to maintain and connect the visual and verbal representations of the story in memory (e.g., Flavell et al., 1986; DeLoache, 2000).

In any case, it seems possible that illustrations could yield benefits in a story reading context in which an adult supports or “scaffolds” children's attention to and understanding of the illustrations. When a parent or other adult reads a story to a child, both the child and the reader may ask questions and make comments about the pictures and text. Research on adult-child story reading suggests that these types of story-reading behaviors enhance children's processing of stories. For example, adult references to print, both verbal and non-verbal, increase preschoolers' references to print (e.g., Justice et al., 2002, 2008). Moreover, parents' attempts to actively engage young children during story reading (Kang et al., 2009), and children's spontaneous utterances to parents (Kim et al., 2011), predict better child story retelling. These types of behaviors during parent-child story-reading also predict children's long-term literacy outcomes, including vocabulary and story comprehension skills (e.g., Whitehurst et al., 1988; Haden et al., 1996; Reese and Cox, 1999; Hood et al., 2008). Although no studies have examined the specific role of illustrations in influencing parent-child story reading interactions, it seems quite possible that illustrations might elicit more discussion than narrative alone, which in turn might enhance children's comprehension and recall of the story. Thus, although illustrations alone might not enhance young children's memories for stories in a controlled story presentation context, illustrations could yield benefits in an interactive story-reading context.

Therefore, we designed this study to examine the specific role of illustrations in influencing parent-child storybook reading behavior and eliciting parent and the effects on 3.5 to 4.5-year-old children's story comprehension and recall. Children in this age range did not benefit from illustrations when they accompanied audio-recorded stories in Greenhoot and Semb (2008). In the current study we tested whether they would benefit when the same story narratives and pictures were used in an interactive story-reading context. We asked parents to read either an illustrated or non-illustrated story to their children, and later asked the children to retell the story to an experimenter. We analyzed the qualities of the story reading interactions in these two conditions and examined the relations to children's story recall. The specific aims were to (a) assess the influence of story illustrations on parent and child story-reading behavior, (b) examine the effects of illustrations on young preschoolers story recall in this interactive story-reading context, and (c) to determine how group and individual differences in parent-child story-reading behavior relate to preschoolers' recall of illustrated and non-illustrated stories.

## Methods

### Participants

The participants were 26 preschoolers (*M* = 48 months, range = 38–58 months) and their primary (or co-primary) caregivers from a small city in the Midwestern United States. Participants were volunteers who were recruited through ads distributed through local preschools, posted in the public library, and published in local newsletters aimed at families. Fifty-eight percent of the children were female. Almost all (92%) of the dyads were Caucasian, 4% were Hispanic and the remaining did not specify the parent or child's ethnicity. The mean level of education of the participants' parents was 16.8 years (range = 13–18 years) for mothers and 15.8 years (range = 7–18 years) for fathers, indicating that the children were generally from college-educated, middle-class families. Responses to a background questionnaire administered at the beginning of the study indicated that none of the child participants knew how to read at the time of the study. One parent-child dyad was unable to schedule the second session within a reasonable time frame, making the sample size for the 1-week recall analyses 25 rather than 26.

### Procedure

The participants met individually with the experimenter for two sessions. To make participant recruitment easier, parents were given the choice of scheduling both sessions in a university laboratory space or in their homes; about half of the participants chose to meet in their homes (for both sessions) instead of the laboratory space, and this variable was relatively evenly distributed across story-reading conditions (39% in the Illustrated condition and 54% in the Non-Illustrated condition, *X*^2^_(1)_ = 0.62, *p* = 0.43). Dyads were randomly assigned to the Illustrated (I) or Non-Illustrated (NI) story-reading condition. The stories and the illustrations were the same as those used in two conditions of our previous study, and were experimentor-designed rather than commercially available books (Greenhoot and Semb, 2008). The Illustrated and Non-Illustrated book versions described the same 18 events involving a fictional animal character; in the Illustrated books, the gist of each event was depicted in two colorful illustrations. The main events of the story described actions of the fictional animal, as described below. Parents were encouraged to “read or tell the story” in the same manner in which they normally read stories to their child.

After the parent finished reading the story to his or her child, he or she completed a demographic questionnaire while the child completed a puzzle as a distracter task (approximately 5 min). This activity was followed by a Memory Interview in which the experimenter asked the child to recall the story. Approximately 1 week later (*M* = 7 days, range = 5–9 days) the same experimenter again met with the dyad and administered a second Memory Interview to elicit the children's delayed recall of the story. We videotaped the story-reading interactions and the memory interviews for later analysis. All procedures in this study were approved by the university Human Subjects Committee.

### Materials

#### Stories

As in the previous study, we used two different stories within each story presentation group to ensure that any effects were not specific to a particular set of stimuli. Within each story presentation group participants were randomly assigned to either the “Basil the Bobbin” story or the “Wilbur the Woozle” story, each of which were constructed for use in this (and other) research in our laboratory. Each story began with a brief description of the characteristics of a fictional type of animal (e.g., woozles have smooth skin, long snouts, fat bellies that they slide around on, and live in hollow logs) and then described a series of 18 events involving a specific animal character. An event was defined as a self-contained set of actions or occurrences that revolve around a central character, time, or place that have independent coherence (Linton, 1986); for these stories, each event was broadly defined by a place or time period in the story. Each event first described the setting for the event and initiating actions (e.g., Wilbur the Woozle sees a beautiful, shiny rock in a hole, but cannot get it out with his paw), and then described response actions and resolution of the event (e.g., Wilbur uses his long snout to push the rock out of the hole). The event narratives in the Bobbin story averaged 6.7 sentences, and 64.7 words, each, whereas those for the Woozle story averaged 6.6 sentences and 61.9 words.

In the Illustrated condition, there were two pictures per event, illustrating the gist of each event component (i.e., the setting/initiating actions and the resolution). For example, for the shiny rock event in the Woozle story, the first illustration depicted Wilbur looking at a shiny rock in a hole. The second illustration showed Wilbur with his snout extended to the hole to push it out.

#### Memory interview

The Memory Interview consisted of a series of open-ended questions about the story at two levels of specificity. The interview began with a very general question (e.g., “What happened in that story about Wilbur the Woozles search for a new home?”) to which children were encouraged to provide as much information as possible. This general probe was followed by more focused open-ended questions about each story event not already recalled by the child (e.g., “What happened when Wilbur saw the shiny rock?”). Children were prompted to elaborate on each event with, “Can you tell me more about that?” or “How did that happen?” All children were interviewed by the same experimenter for both sessions.

### Coding

#### Story-reading interactions

Drawing on previous research on parent-child story reading, we coded the videotaped story-reading interactions for a number of qualities, which are summarized in Table 1. First, we coded the frequency of several types of parent and child extra-textual comments and non-verbal behaviors during reading. All comments were divided into propositions (subject-verb constructions), with each unique or non-redundant proposition scored as a comment. We categorized these comments and behaviors according to their relation to the book content, including direct references to the book and/or the pictures or text in the book, references to the events described or depicted in the book, and elaborations on story content. We also coded for child inattentiveness by identifying the number of story events during which the child was inattentive to the story, and counted the number of parent attempts to redirect children's attention to the story reading task. We also scored the interactions on several global qualities. First, raters made a yes-no judgment about whether the story-reading activity was highly “interactive,” defined as involving susbtantial verbal and/or non-verbal exchange between parent and child. They also made yes-no judgments about whether the parent was engaged in the story-reading task, and yes-no judgments about whether the child was largely distracted throughout the story-reading session. Finally, they rated parent emotional expressiveness on a 0–2 scale, with a 0 being no emotion expressed, a 1 being occasional or intermittent displays of emotion, and a 2 being consistent expression of emotion. All coding was completed by two raters. Interrater reliability, calculated on approximately 20% of the videotaped interactions, was high, with the raters agreeing on 93% of the scores they assigned. In addition to the interaction coding, the raters measured the length of time parents and children took to read each book.

**Table 1:** **Story-reading interaction codes**.

**Type of code**	**Coding category**	**Description**	**Scoring**
Specific comments and behaviors	Parent or child book-directed behaviors	Pointing to book content (text or pictures), labeling, describing or otherwise discussing pictures or words presented in the book (e.g., “What is he doing now?”)	Frequency
	Parent or child event-related comments	Comments or questions directly related to the story events, such as inferences about causality (e.g., “He must have been hungry”) or predictions about what will happen (e.g., “Now he's going to get breakfast”)	Frequency
	Parent or child elaborations	Comments or questions that involved relating the story to pre-existing knowledge (e.g., “What other animals eat bugs?”), to one's own life (e.g., “Grandpa's name is Wilbur, too”), or evaluative remarks (e.g., “Flies are yucky.”)	Frequency
	Child inattention	Indicators that the child is not attending to the story	Number of story events
	Parent redirections	Parent comments intended to redirect the child's attention to the story	Frequency
Global qualities	Interactive story-reading	Substantial verbal and/or non-verbal exchange between parent and child	0 = no
			1 = yes
	Child distraction	Child highly disengaged; no attempts to interact with parent about the story and does not respond to parent attempts	0 = no
			1 = yes
	Parent engagement	Parent is engaged in story reading task, makes consistent attempts to go beyond text-reading and respond to child attempts	0 = no
			1 = yes
	Parent expressiveness	Degree of parent emotional expressiveness during story reading	0 = none
			1 = intermittent
			2 = consistent

#### Story recall coding

Children's recall performance was evaluated for accuracy and completeness by comparing their reports in the memory interviews to the actual text of the stories. Each story event that the child reported was assigned one of four codes: Accurate Complete, Elaboration, Partial Recall, or Distortion. If the child recalled the gist of what was stated in the text about an event, the response was coded as Accurate Recall (e.g., “Wilbur got the rock out of the hole with his snout.”). Accurate Recall also included embellishments on the text that were generally consistent with the text (e.g., “Wilbur dug and dug with his snout until he finally knocked the rock out of the hole.”). If a child only recalled part of an event (i.e., if the child did not report the event resolution) but was otherwise accurate, the event was assigned a Partial Recall code (e.g., “Wilbur could not get the rock out with his foot.”). A Total Recall score was calculated as the sum of Accurate Recall and Partial Recall. A Distortion was coded when a child distorted a story event in recall (e.g., “Wilbur ate the rock.”), whereas an Intrusion was coded when a child reported an event that was not described in the text at all (e.g., “Wilbur rode a bicycle.”). Two research assistants each scored the interview transcripts, overlapping on approximately 20% of the sample for reliability purposes. Interrater reliability for the memory codes was quite good, as indicated by percent agreement of 94%.

For each child at each interview, we calculated a Total Recall score as the proportion of story events receiving an Accurate Complete, Elaboration, or Partial Recall code. We also calculated a more conservative recall score of Accurate Complete Recall, consisting of the proportion Accurate Complete and Elaboration codes. Finally, we calculated Recall Error scores as the proportion of story events for which children received Distortion or Intrusion scores.

## Results

We designed the analyses to address each of the three major aims of the study. First, we examined group differences in parent and child story-reading codes, to determine whether the illustrations affected the quality of parent-child storybook reading. Next, we tested the effects of the illustrations on children's story recall. Third, we explored how individual differences in parent-child story reading interactions related to children's story recall in the two presentation conditions.

### Parent-child story-reading behavior

Preliminary analyses indicated that parent comments, summed across the individual story reading codes, were quite frequent (*M* = 22.5) and more than twice as common as comments from the child (*M* = 10.8). Individual parent story-reading codes were interrelated; parent book-related comments were positively associated with parent event-related comments and parent redirections (*r*s ≥ 0.48, *p*s ≥ 0.01), and parent elaborations were correlated with parent event-related comments and parent emotion expressiveness (*r*s ≥ 0.65, *p*s ≤ 0.0003). Child story reading codes were also interrelated, with child event-related comments positively associated with book-related comments and elaborations (*r*s ≥ 0.61, *p*s ≤ 0.0009). Finally, parent and child measures also correlated with each other, with particularly strong associations between parent and child codes for the same types of comments (*r*s ≥ 0.57, *p*s ≤ 0.002).

There were several indications that the illustrations affected parent-child story-reading interactions. As shown in Table 2, both parents and children made more direct references to the book and more comments about the text in the Illustrated than Non-Illustrated condition. In contrast, illustrations did not produce significant differences in the frequency with which parents or children made comments about the story events or elaborated on story content. Furthermore, although parents tended to do more redirecting and children were inattentive more frequently in the Non-Illustrated condition, these differences did not reach significance. Interestingly, parent emotion expressiveness scores were higher in the Non-Illustrated condition than the Illustrated condition.

**Table 2:** **Mean scores (and standard deviations) of parent and child story-reading codes, by condition**.

	**Illustrated condition**	**Non-illustrated condition**	***t*_(24)_**	***p*-value**
**PARENT BEHAVIORS**				
Book-directed behaviors	12.4 (16.2)	2.5 (45)	2.13	0.044
Event-related comments	8.8 (8.9)	8.6 (9.0)	0.07	0.94
Elaborations	4.6 (6.0)	7.1 (8.3)	−1.16	0.26
Parent redirections	1.2 (2.3)	0.5 (0.5)	1.19	0.25
Parent emotional expressiveness	0.85 (0.69)	1.54 (0.52)	−2.89	0.008
**CHILD BEHAVIORS**				
Book-directed behaviors	5.3 (6.9)	0.8 (2.3)	2.26	0.033
Event-related comments	7.0 (7.9)	4.7 (5.3)	−0.88	0.39
Elaborations	1.7 (1.7)	1.6 (1.7)	0.58	0.57
Inattention	3.9 (3.2)	6.5 (6.9)	−1.21	0.24

**Figure 1:**
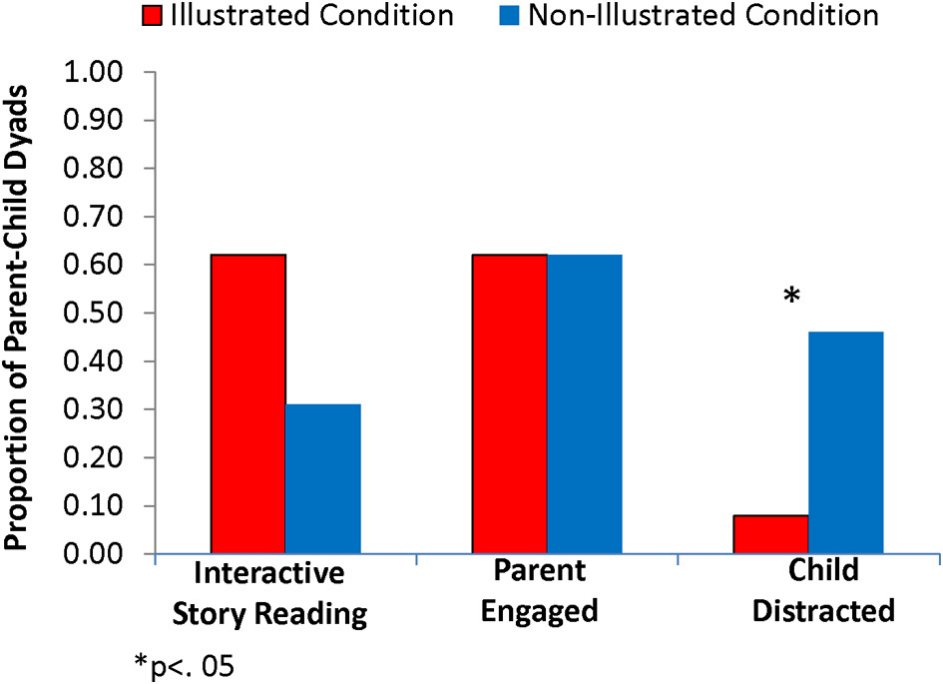
**Proportion of parent-child story-reading sessions scored as Interactive, Parent Engaged, and Child Distracted, by condition**.

Figure 1 summarizes the global story reading interaction scores for the two conditions. Almost two-thirds of reading sessions in the Illustrated condition were coded as Interactive story-reading, compared to 31% in the Non-Illustrated condition, although the difference did not reach significance, *X*^2^_(1)_ = 2.48, *p* = 0.12. Although parent engagement was unaffected by Illustration condition, children in the Non-Illustrated condition were significantly more likely to be distracted from story reading than those in the Illustrated condition, *X*^2^_(1)_ = 4.89, *p* = 0.027. Indeed, of the 7 children rated as highly distracted, 6 were in the Non-Illustrated condition. Further, 4 of these children (all Non-Illustrated) had parents who made many attempts at redirection or engagement (i.e., between 19 and 60 parent comments and gestures across the story reading session).

There was no significant difference between conditions in the length of the reading sessions, *t*_(24)_ = 0.58, *p* = 0.57. Book reading took an average of 9 min 18 s in the Illustrated condition, and 9 min 48 s in the Non-Illustrated condition.

### Illustrations and children's story recall

**Figure 2:**
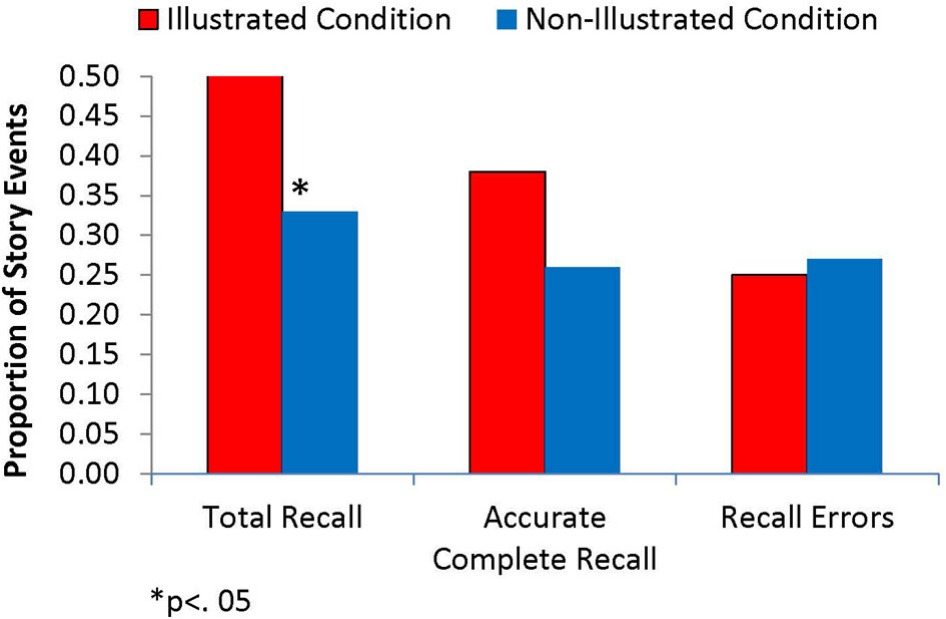
**Recall performance at the immediate interview, by story-reading condition**.

**Figure 3:**
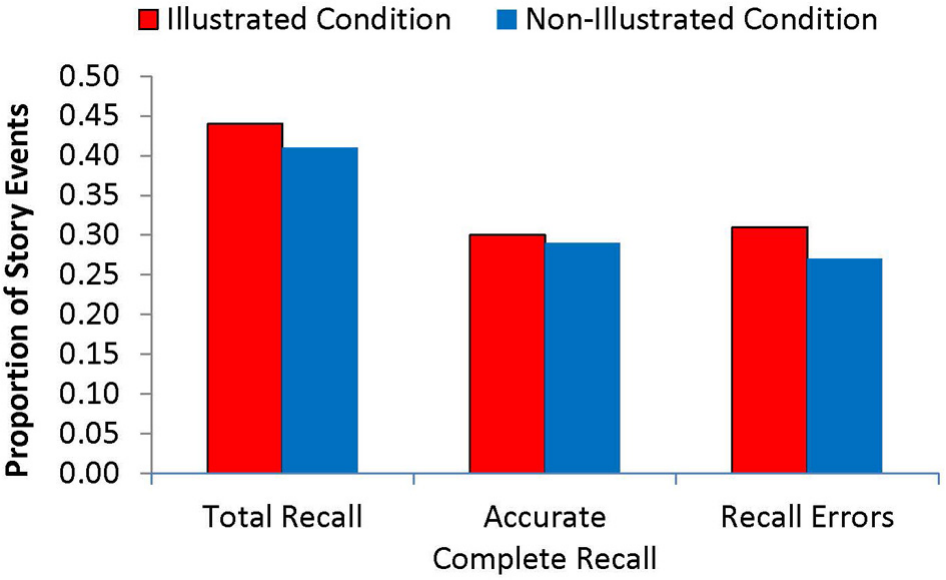
**Recall performance at the 1-week interview, by story-reading condition**.

Figure 2 shows the Total Recall, Accurate Complete, and Recall Error scores for the immediate interview, as a function of story presentation condition. Figure 3 shows a parallel set of scores for the 1-week interview. Even according to the more liberal scoring scheme used for calculating Total Recall, the children remembered less than half of the events depicted in the story at both interviews. Furthermore, a little over one fourth of the story events were recalled incorrectly at both interviews. Thus, this recall task seemed to be a challenging one for the participants.

Importantly, however, children's story recall was notably better in the Illustrated condition than the Non-Illustrated condition. Specifically, children in the Illustrated condition had higher Total Recall scores than those in the Non-Illustrated condition at the immediate interview, *t*_(24)_ = 2.14, *p* = 0.043. Although Accurate Complete Recall was also higher in the Illustrated condition at the first interview (38% vs. 26%), this difference did not reach significance, *t*_(24)_ = 1.52, *p* = 0.14. The advantage in Total Recall for the Illustrated condition over the Non-Illustrated condition was somewhat maintained at Time 2 (44% vs. 41%), but the difference was no longer statistically significant. Recall Error scores did not differ between story presentation groups at either interview. Thus, in contrast to our previous findings with prerecorded stories in the same age group, illustrations in parent-presented stories increased children's abilities to remember the story narrative, particularly in the short term.

### Individual differences in parent-child story reading and children's story recall

The goal of these anlayses was to examine how the qualities of parent-child story reading predicted children's abilities to retell the story later. We were also interested in whether the qualities of parent-child story reading could explain the modest improvements in recall performance produced by the story illustrations.

#### Identifying covariates

First, to identify possible covariates to be included in our individual difference analyses, we used pearson correlations, *t*-tests and chi-square analyses to test whether the variables of interest (story reading measures and story recall measures) were associated with variables such as the children's age (in months), gender, and reading experience, mother's years of education, material set, and experiment location (lab vs. home). None of the story reading measures were associated with any of these variables. Children's story recall was related to their age and the location of their experimental sessions. Specifically, older children had higher Accurate Complete Recall and Total Recall scores at both interviews (*r*s ≥ 0.42, *p*s ≤ 0.031), and lower Recall Error scores at both interviews (*r*s ≤ −0.38, *p*s ≤ 0.06), although the association with 1-week Recall Errors was only marginally significant. Children who participated in a lab setting had superior story recall to those who participated in their homes, *t*s > 2.79, *p*s < 0.01. For instance, Total Recall scores at the immediate interview averaged 0.52 in the lab condition and 0.30 in the home condition. Story recall was unrelated to child gender, material set, or mothers' education. Therefore, we controlled for both session location (lab = 1; home = 0) and child age (in months) in these analyses.

#### Predictive models

We tested the relations between story-reading qualities and story recall in a series of general linear models (GLMs) predicting each of the recall measures (Total Recall, Accurate Complete Recall, and Recall Errors) at the immediate and 1-week interview. This approach enabled us to control for age and location and to test each parent story reading code along with the corresponding child code(s). We ran the GLMs separately for memory measures at the two different time points because a traditional repeated measures approach would have resulted in casewise deletion of data from the child who did not participate in the second memory interview.

To test whether the story-reading qualities explained group differences in recall, we first ran a set of models containing only location, age, and group (Model 1). By comparing the parameter estimates for group in this simple model to those in models containing story-reading variables, we could assess the degree to which the story-reading qualities accounted for group recall differences. The small sample size limited the number of predictors we could include in any one model, therefore we tested several sets of models, each with a different category of story reading codes as additional predictors (see Table 3). The second set of models (Model 2) tested the predictive value of parent and child event-related comments, over and above group, age, and location. Likewise, the set of models labeled Model 3 tested the predictive values of child inattention and parent redirection when group, age and location were in the model. Finally, model 4 tested the predictive value of the global story reading qualities (i.e., parent emotion expressiveness, parent engagement, child distraction, and interactive story reading). The models with the categories of book-related comments and elaborations as predictors revealed no links between these variables and story recall, therefore we do not present them here.

**Table 3:** **Parameter estimates (and standard errors) generated by GLMs predicting recall performance from child age, experiment location, group, and parent-child story reading codes**.

	**Immediate interview**			**1-week interview**		
	**Total recall**	**Accurate complete**	**Recall errors**	**Total recall**	**Accurate complete**	**Recall errors**
**MODEL SET 1**						
Group (Illustrated)	**0.13^b^ (0.09)**	0.08 (0.07)	0.03 (0.08)	−0.028 (0.063)	−0.053 (0.063)	0.09 (0.08)
Age	0.01 (007)	0.012 (0.007)	0.017^*^ (0.007)	**0.012^b^ (0.006)**	0.020^**^ (0.006)	**0.014^b^ (0.008)**
Lab	**0.17^b^ (0.08)**	**0.17^b^ (0.08)**	0.027 (0.08)	0.30^***^ (0.07)	0.20^**^ (0.066)	−0.07 (0.09)
t**MODEL SET 2**						
Group (Illustrated)	**0.15^b^ (0.07)**	0.10 (0.08)	0.021 (0.08)	−0.038 (0.07)	−0.053 (0.067)	0.09 (0.09)
Age	0.012^**^ (0.007)	0.014^**^ (0.006)	−0.016^**^ (0.007)	**0.012^a^ (0.006)**	0.021^**^ (0.006)	−0.013 (0.008)
Lab	**0.15^b^ (0.08)**	**15^b^ (0.07)**	0.013 (0.084)	0.28^***^ (0.07)	0.18 ^**^ (0.07)	−0.088 (0.096)
Parent event-related	0.013^*^ (0.007)	0.015^**^ (0.006)	0.008 (0.007)	−0.000 (0.006)	0.005 (0.006)	0.004 (0.008)
Child event-related	−0.009 (0.009)	−0.011 (0.009)	−0.005 (0.010)	0.004 (0.008)	−0.001 (0.008)	−0.001 (0.01)
t**MODEL SET 3**						
Group (Illustrated)	0.10 (0.084)	0.035 (0.078)	0.06 (0.077)	−0.09 (0.071)	−0.035 (0.069)	0.09 (0.093)
Age	0.009 (0.008)	0.012 (0.008)	−0.011 (0.007)	0.02^*^ (0.007)	0.01 (0.007)	−0.008 (0.009)
Lab	**0.17^b^ (0.089)**	**0.16^a^ (0.083)**	−0.037 (0.082)	0.20^*^ (0.074)	0.32^*^ (0.073)	−0.13 (0.097)
Parent redirects	0.023 (0.026)	0.035 (0.024)	−0.11 (0.024)	0.03 (0.022)	−0.005 (0.021)	0.02 (0.028)
Child inattention	−0.005 (0.009)	−0.006 (0.008)	0.018^*^ (0.008)	−0.005 (0.007)	−0.005 (0.007)	0.01 (0.009)
t**MODEL SET 4**						
Group (Illustrated)	0.11 (0.11)	0.038 (0.10)	−0.14 (0.11)	0.004 (0.081)	−0.018 (0.086)	−0.05 (0.11)
Age	0.004 (0.01)	0.007 (0.009)	−0.02^*^ (0.009)	**0.014^a^** (0.007)	0.024^*^ (0.007)	−0.013 (0.010)
Lab	0.21^*^ (0.08)	0.20^*^ (0.078)	0.03 (0.083)	0.31^*^ (0.063)	0.20^*^ (0.066)	−0.10 (0.089)
Parent emotion exp.	0.004 (0.076)	0.017 (0.072)	−0.16^*^ (0.076)	**0.12^b^ (0.062)**	0.11 (0.066)	**−0.17^b^ (0.088)**
Parent engagement	0.24 (0.15)	0.017 (0.14)	0.006 (0.15)	−0.11 (0.11)	−0.06 (0.12)	0.008 (0.16)
Child distraction	**−0.28^a^ (0.14)**	**−0.26^a^ (0.13)**	−0.06 (0.14)	−0.03 (0.10)	−0.008 (0.11)	0.06 (0.15)
Interactive story reading	−0.24 (0.17)	−0.14 (0.16)	0.10 (0.17)	0.07 (0.13)	0.08 (0.13)	0.22 (0.18)

*** p < 0.001;

** p < 0.01;

* *p < 0.05;*

a p < 0.07;

b *p < 0.10*.

Table 3 displays the parameter estimates (and standard errors) generated by the GLMs. In Model 1, participation in the lab setting and child age were all positively related to measures of children's recall performance. Furthermore, being in the Illustrated group was related to better Total Recall at the first interview, although this effect was only marginally significant. But the results from Models 2 through 4 provide some evidence that extra-textual comments and behaviors during story reading contribute to children's story recall, over and above the effects of the Model 1 variables. The GLMs testing parent and child event related comments showed that parent comments (Model 2) were related to both Total Recall and Accurate Complete Recall at the immediate interview. The more parents discussed the events in the book with their children, the better children remembered the story events after a short distracter. Importantly, however, parent event-related comments do not appear to explain the advantage in Total Recall of the Illustrated group, as the parameter estimate for group was not reduced by the inclusion of event-related comments in Model 2.

The models including child inattention and parent redirects (Model 3) revealed that child inattention predicted more Recall Errors at the first interview. Therefore, the more story events for which children were not attentive, the more errors they made in retelling the story. Finally, the models that included the global qualities (Model 4) showed that higher parent expressiveness predicted fewer recall errors at the immediate interview and recall of more story events at the delayed interview. Additionally, children who were completely disengaged during story reading had lower levels of Total Recall and Accurate Complete Recall at the immediate interview. Interestingly, in this last set of models the parameter estimate for group was somewhat reduced, suggesting that increased rates of child disengagement in the Non-Illustrated condition could be at least partially responsible for the recall advantage seen in the Illustrated condition.

## Discussion

Illustrations are commonplace in storybooks for young children, yet the scant research on their influence on children's story retelling has suggested that young preschool children actually learn very little from story illustrations when they are presented in a non-interactive story-reading context (e.g., Greenhoot and Semb, 2008). The results of this investigation suggest that illustrations do enhance young preschoolers' story recall when they are presented in an interactive story reading context. Thus, young preschoolers may be unable to glean both visual and auditory information from a story and/or integrate the information in a context that requires them to do this alone, but our current findings are consistent with the view that parents help support or “scaffold” such processes in an interactive story reading context. Parents may use the pictures to keep children engaged in the reading activity, help children see the relevance of the illustrations for comprehending the story, and/or facilitate children's ability to attend to the pictures and verbal information and integrate them in memory. These findings fit well with sociocultural models of development, which highlight the role of adult-child interaction in guiding and supporting children's participation in activities and socializing their skill development (Vygotsky, 1978; Rogoff, 1990; Nelson and Fivush, 2004). It should be noted, however that the parent-supported enhancements observed in this study were not especially robust or long-lasting; pictures facilitated children's immediate recall of the stories but significant benefits were not maintained after a 1-week delay.

Our analyses of the story reading interactions reveals some of the things parents might do to support children's processing of the illustrations. These analyses showed that the presence of illustrations influenced both parent and child behaviors during story reading. Both parents and children made more references to the book in the Illustrated condition, and this increase in book references was not limited to the illustrations themselves. Parents and children also made more references to the text when pictures were present than when they were not. Nonetheless, the predictive models showed that these references to the book were not directly related to children's recall of the story events.

The global measures of story reading quality also indicated that the illustrations prompted a more interactive and engaged style of story reading. Children in the Illustrated condition were less likely to be rated as distracted than those in the Non-Illustrated condition. Indeed, of the 7 children who were rated as distracted, 6 were in the Non-Illustrated condition and 4, all of whom were in the Non-Illustrated condition, had parents who had made numerous engagement attempts. Thus, these parent efforts at engagement may have failed without the support of pictures. The predictive models showed that children who more frequently displayed inattentiveness, and those who were judged to be completely distracted during the story presentation, had poorer recall performance at the immediate interview. Moreover, the predictive value of group was somewhat reduced when these attention variables were included in the models. These patterns suggest that the parents may have used the illustrations to hold children's attention to the story reading activity, leading to improved recall of the story events, at least in the short-term.

Interestingly, parent emotional expressiveness was actually higher in the Non-Illustrated condition than the Illustrated condition, suggesting that parents may have tried to compensate for the absence of illustrations by increasing their emotional expressiveness as they read. Perhaps they were using this elevated emotional expressiveness to keep children engaged with the story reading activity. Thus, our small sample of well-educated mothers appears to be quite sensitive to the story reading context. The fact that parent emotion expressiveness also was related to fewer recall errors at both interviews and higher overall recall at the 1-week interview recall suggests that compensation attempts for the absence of pictures could have reduced the robustness of the differences between the Illustrated and Non-Illustrated conditions. Of course, within-person comparisons of maternal story reading with illustrated and non-illustrated stories would provide a more definitive evaluation of this claim. It also remains to be seen whether this pattern would generalize to a more diverse sample, as pre-emergent reading behaviors have been shown to vary according to socioeconomic status (Bus et al., 1995). It would also be important to explore whether these illustration effects on parent expressiveness would generalize to different types of stories (e.g., stories that are either more or less interesting than those used here).

References to the events described in the story were the one category of story-reading measures that did not differ across the Illustrated and Non-Illustrated groups. However, consistent with the literature on parent-child story-reading interactions (e.g., Kang et al., 2009; Kim et al., 2011), individual differences in this dimension of story reading interactions were linked to differences in children's recall at the initial interview. In particular, children whose mothers offered more comments and questions about the events described in the stories, such as inferences or predictions about what will happen, learned more from the stories than other children. Therefore, although this feature of the interactions did not seem to account for the illustration effects, it seemed to support children's recall of the events in the story regardless of the presentation format.

Overall, the pattern of results in this study suggests that the illustrations prompted more interactive story reading and more behaviors known to predict improved literacy outcomes for children (e.g., Whitehurst et al., 1988; Haden et al., 1996; Reese and Cox, 1999; Hood et al., 2008). Furthermore, the illustrations did produce recall enhancements and parent and child story-reading behaviors predicted children's story recall. Nonetheless, our story reading measures only partially accounted for the effects of the illustrations on children's story recall. This pattern suggests that our story reading measures simply may not have captured the actual mediating variables. For instance, it seems likely that it is simply the joint attention established between parent and child in this context supports children's processing of the illustrations. It is also quite possible that the dimensions of story-reading that we measured support dimensions of children's processing and recall of the stories that we did not capture in this study.

The current study was limited to mother-child story storybook reading, and there is some evidence that parent-child book-reading may differ for mothers and fathers (Anderson et al., 2004). For instance, Anderson et al. (2004) found that fathers asked for more clarification and made more confirmations when reading informational books to their 4 year-olds than mothers. Although previous research has not found differences in how fathers and mothers read narrative stories to children, future research should explore whether the effects of illustrations observed in mothers in this study extend to fathers' reading styles.

Another future consideration in understanding illustration effects on book reading with young children is the genre of book, as some work has documented differences in book reading interactions depending on genre of book (Mason et al., 1989; Price et al., 2009). Price et al. (2009) found substantial differences in parents' book reading, for storybooks verses informational, non-fiction books. For instance, parents spent more time reading and commenting on the informational books than the storybooks. Moreover, parents provided more feedback to the child, commented more about the character/animal, and made more elaborations and inferences during the informational book reading. Interestingly, the storybooks in their study averaged eight more illustrations than the informational books. Similar interactional patterns have been observed with teacher book sharing (Mason et al., 1989). Parents' and teachers' heightened commenting and elaborating on informational books could potentially provide even greater scaffolding for illustration processing, leading to heightened picture facilitation effects.

These findings have a number of implications for understanding the optimal ways of presenting stories to young children, and address issues that are particularly important in an age of increasing reliance on non-human sources of information for children. First, this study highlights the fact that a story presentation context that allows for social exchange may be critical for helping children to process and retain narratives or other material from storybooks. Second, for young preschoolers in the prereading phase, story illustrations help to elicit the sorts of story-reading interactions that are known to enhance children's story processing and relate to positive literacy outcomes. One necessary extension of this line of work is to examine how digital technology affects shared book reading. Some work suggests that children adopt a more participatory role when read an e-book by a researcher than a traditional storybook (Moody et al., 2010). Yet other work suggests that touch sensitive e-readers may negatively impact parent-child shared book reading interactions and children's comprehension (Parish-Morris et al., 2013). With the proliferation of e-readers for people of all ages, it is time to find out whether such media can provide as supportive an environment for adult-child story-sharing interactions as a traditional illustrated storybook.

### Conflict of interest statement

The authors declare that the research was conducted in the absence of any commercial or financial relationships that could be construed as a potential conflict of interest.
